# Antioxidative Categorization of Twenty Amino Acids Based on Experimental Evaluation

**DOI:** 10.3390/molecules22122066

**Published:** 2017-11-27

**Authors:** Naijin Xu, Guanqun Chen, Hui Liu

**Affiliations:** College of Medical Laboratory, Dalian Medical University, Dalian 116044, China; naijinxu@aliyun.com (N.X.); guanqungq@163.com (G.C.)

**Keywords:** amino acid, total antioxidant capability, redox titration

## Abstract

In view of the great importance bestowed on amino acids as antioxidants in oxidation resistance, we attempted two common redox titration methods in this report, including micro-potassium permanganate titration and iodometric titration, to measure the antioxidative capacity of 20 amino acids, which are the construction units of proteins in living organisms. Based on the relative intensities of the antioxidative capacity, we further conducted a quantitative comparison and found out that the product of experimental values obtained from the two methods was proven to be a better indicator for evaluating the relative antioxidative capacity of amino acids. The experimental results were largely in accordance with structural analysis made on amino acids. On the whole, the 20 amino acids concerned could be divided into two categories according to their antioxidative capacity. Seven amino acids, including tryptophan, methionine, histidine, lysine, cysteine, arginine and tyrosine, were greater in total antioxidative capacity compared with the other 13 amino acids.

## 1. Introduction

Oxidation resistance, as a normal, unavoidable and indispensable physiological process, plays a positive role in maintaining homeostasis in living organisms. However, when oxidation resistance surpasses the organism’s immune burden, it will definitely bring detrimental consequence to the organism, even triggering various chronic, even life-threatening diseases. To counteract the over-oxidation imposed by different kinds of internal and external factors and adapt to the complicated and changeable environment, all the living organisms, especially higher animals like human beings, have gradually developed various fine-tuning anti-oxidizing systems in the long history of evolution. To date, we clearly know a variety of anti-oxidizing systems present in living organisms, and they include some enzymes, such as superoxide dismutase, catalase and glutathione peroxidase, etc. [1,2,3], various proteins, such as albumin, ceruloplasmin, ferritin, etc. [4,5,6], many compounds of relatively small molecules, such as ascorbic acid, α-tocopherol, *β*-carotene, ubiquinol-10, glutathione (GSH), methionine, uric acid, bilirubin [7,8,9] and hydroxytyrosol, etc. [10,11], and some hormones, such as estrogen, angiotensin and melatonin, etc. [12,13,14].

Admittedly, the vital important role of antioxidants in healthcare and disease prevention lead many researchers to the investigation of the antioxidative capacity of various bioactive substances, such as plants [15], animal proteins [16] and several amino acids [17]. Amino acids, to be accurate, alpha-amino acids, the building blocks of proteins in living organisms, naturally attract researchers’ attention. Mostly, the amino acids in vivo exist in proteins, but they are also present in our body fluids as free forms [18]. Therefore, studying the antioxidative activity of amino acids will deepen the understanding of what role these life-sustaining substances play in oxidation resistance.

Although structural analysis may probably arrive at a rough estimation on the antioxidative capacity of amino acids, it is necessary to carry out quantitative studies on all those amino acids found in the body to accurately sort these amino acids in terms of antioxidative capacity. In the present study, we have employed two methods to make a preliminary quantitative analysis of the antioxidative activity of 20 amino acids. Based on this study, it is possible to deduce the antioxidative capacity of certain peptides and proteins.

## 2. Materials and Methods

### 2.1. Reagents and Instruments

Potassium permanganate, sodium oxalate, iodine, potassium iodide, potassium dichromate and sodium thiosulfate were of analytical grade, and the 20-amino acid kit was obtained from Accustandard (New Haven, CT, USA). Using the molecular weight data for the amino acids, as supplied (Table 1), solutions were prepared with distilled water to give amino acid concentrations of 0.5 mol/L.

### 2.2. Micro-Potassium Permanganate Method

The micro-potassium permanganate method was used to measure total antioxidant capacity (AOC) according to the literature [19]. The potassium permanganate solution was prepared by firstly dissolving 0.316 g of potassium permanganate in 50 mL of distilled water, and then, the mixture was further diluted to 100 mL in a volumetric flask. The solution prepared was stored in a brown reagent bottle. The potassium permanganate solution was standardized with standard sodium oxalate solution. The actual concentration of KMnO_4_ solution was 0.019 mol/L; this was diluted to 0.005 mol/L to give the working solution.

Each amino acid solution of 0.5 mol/L was diluted in a ratio of 1:10 with distilled water to give 0.05 mol/L. This was repeated to give a series of solutions with five concentrations: 5 × 10^−2^, 2.5 × 10^−2^, 1.25 × 10^−2^, 6.2 × 10^−3^ and 3.125 × 10^−3^ mol/L.

Twenty microliters of each dilution were transferred to a 96-well microtiter plate; 80 μL of a 0.005 mol/L KMnO_4_ solution were then added to each well in the plate. The plate was put in an oscillator for 15 s to make sure that each mixture was homogeneous. The plate was then put in a water bath at 37 °C for 30 min. Then, OD measurements were performed using a microplate spectrophotometer. The wavelength selected was 550 nm.

**Figure 1:**
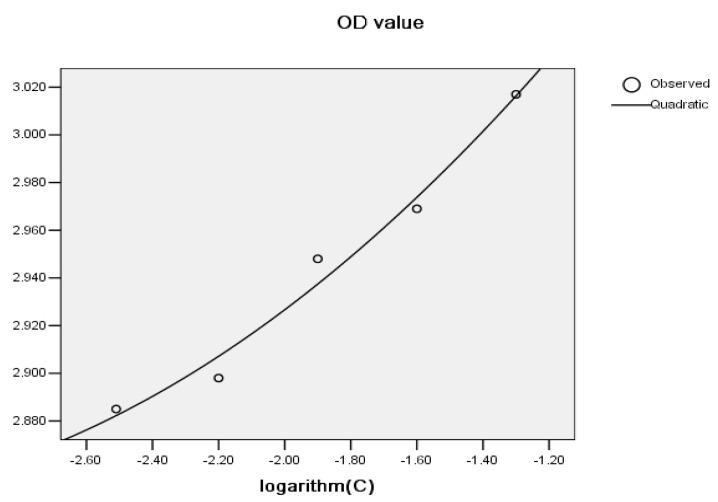
Regression curve of one sample. Where X represents the logarithm of sample dilution times, while Y represents the OD value.

Each sample, with five different concentrations, gave five OD values as shown Figure 1. With five OD values available, it was better to select the area under curve (AUC) to give an assessment of the total AOC as follows: 

A graph including the logarithm of the concentration of an amino acid (−1.30, −1.60, −1.90, −2.20 and −2.50) on the X-axis versus OD values on the Y-axis was plotted (Figure 1). The regression curve obtained by the Statistics software (SPSS) 15.0 package (SPSS Inc., Chicago, IL, USA, 2006) is presented in Figure 1.

The quadratic regression equation obtained was Y = 0.032X^2^ + 0.234X + 3.266, where a = 0.032, b = 0.234, c = 3.266.

The integral formula below was applied to calculate the AUC, which represented the antioxidative capacity of substances reacting with permanganate.

$$\int_{k_{1}}^{k_{2}}{\mathrm{ax}}^{2}+\mathrm{bx}+\mathrm{cdx}=\frac{a}{3}x^{3}+\frac{b}{2}x^{2}+\mathrm{cx}=\;(1/3)\;\times \;a(k_{2}^{3}\;-\;k_{1}^{3})\;+\;(1/2)\;\times \;b(k_{2}^{2}\;-\;k_{1}^{2})\;+\;c(k_{2}\;-\;k_{1})$$

where k_1_ and k_2_ were −2.51 and −1.30, representing the logarithm of the lowest and highest dilutions, respectively. The substitution of the values of a, b and c into the equation resulted in a value of 3.558, which represented the area under the curve. If R^2^ < 0.95, the area was calculated by the ladder-shaped method [19]. The AUC was in inverse proportion to AOC.

### 2.3. Micro-Iodometric Method

The micro-iodometric method was also used to measure total AOC according to the literature [19]. Following the same procedure for preparing potassium permanganate solution, 100 mL of iodine solution (0.05 mol/L) were prepared and then diluted to a concentration of 0.0015 mol/L. The prepared solution was stored in a brown reagent bottle. Indirect titration was employed for the standardization of the iodine solution, using standard potassium dichromate and sodium thiosulfate.

In the same way, 100 mL of starch solution (0.005 g/mL) were prepared.

For the inverse titration method, 0.0015 mol/L iodine solution were diluted to five different concentrations by arithmetic dilution. Initially, 100, 80, 60, 40 and 20 μL of iodine solution were added to five test tubes followed by 0, 20, 40, 60 and 80 μL of distilled water, respectively, to give iodine solutions of 1.5 × 10^−3^, 1.2 × 10^−3^, 0.9 × 10^−3^, 0.6 × 10^−3^ and 0.3 × 10^−3^ mol/L.

To each of the five test tubes, an equal amount of starch solution was added, so the original concentrations of iodine solution were halved. Eighty microliters of each mixed solution were added to the wells of a 96-well microtiter plate. 

Each amino acid solution of concentration 0.5 mol/L was diluted to 0.05 mol/L. Twenty microliters of the sample to be measured were added to each well in the microtiter plate. The plate was then oscillated for 15 s, to ensure that each mixture was homogeneous. The plate was then placed in a water bath at 37 °C for 30 min.

OD measurements were performed using a Microplate Spectrophotometer. The wavelength selected was 570 nm. As described for the micro-potassium permanganate method, the AUC was also in inverse proportion to the AOC.

### 2.4. Quantitative Analysis

The test with each sample was repeatedly performed three times, and then, the median value obtained from the three tests was taken as the observed value. The product of the experimental data obtained by the two methods was considered as a quantitative index to analyze the antioxidative ability. A lower value sample was considered as a stronger antioxidative activity. Experimental data were ranked by quantitative index. A graph including the rank number on the X-axis versus quantitative index on the Y-axis was plotted to sort twenty amino acids based on quantitative index of AOC.

### 2.5. Statistical Analysis

The relationship between the results from the two methods was assessed by the correlation test. The analysis was conducted using SPSS statistical analysis software. *p*-Values less than 0.05 (two-tailed) were considered to be statistically significant.

## 3. Results

**Figure 2:**
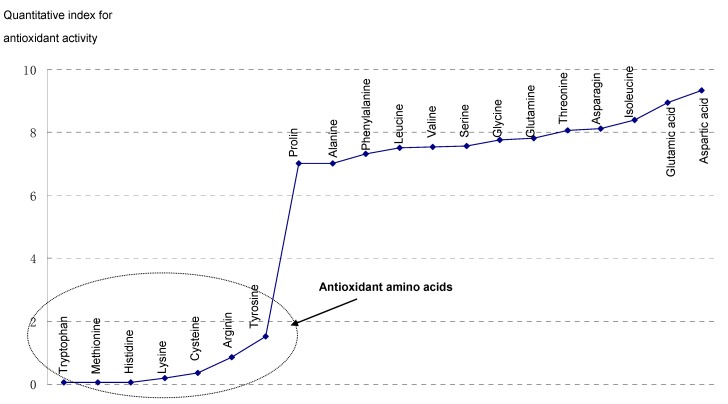
Categorization of twenty amino acids based on experimental evaluation. The value of quantitative index is inversely proportional to antioxidant activity.

The results in Table 2 show that the detection results from the two method were highly correlated (r = 0.869, *p* < 0.001). Different amino acids had different antioxidative activities; seven amino acids, including tryptophan, methionine, histidine, lysine, cysteine, arginine and tyrosine, had strong antioxidative activity, where the others had lower or almost no antioxidant activity, as shown in Figure 2.

## 4. Discussion

As stated in the Introduction, a greatly integrated antioxidative system exists in the body [20]. Under normal circumstances, the free radicals in the body are always in dynamic equilibrium with the antioxidative system [21]. Since serum albumin plays an important role in antioxidation [22,23], to simulate the real scenario in vivo, the concentrations of the amino acids in the experiments were set to be the total level of human plasma protein (the concentration of human serum protein is 70 g/L). The mean concentration of the 20 amino acids used in our research was appropriately 0.5 mol/L, and this could provide a sensible comparison. 

The oxidation processes of amino acids involve very complex kinetics and mechanisms. The following is a brief elucidation for some amino acids.

The group of amino acids we studied is the so-called α-amino acids. They all contain an amino group (NH_2_), a carboxyl group (COOH), a hydrogen (H) and a side chain (R), with NH_2_ attached to the α-carbon, which is next to the COOH. At physiological pH values, these amino acids exist mainly as dipolar ions (or zwitterions) [24]. Due to structural differences in the side chains, the antioxidation mechanisms and capacity of these amino acids vary greatly. 

**Figure 3:**
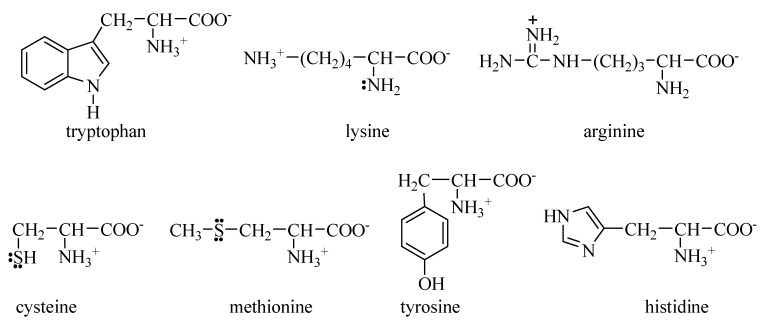
Amino acids with strong antioxidant ability.

There are seven amino acids with strong antioxidative capacity, whose structures are shown in Figure 3.

Amino acids with electron-rich aromatic rings in side chains, e.g., tryptophan and tyrosine, are easily oxidized. Because the tryptophan molecule contains a pyrrole ring, with five ring atoms (four carbon atoms and one nitrogen atom) sharing six electrons, it is susceptible to oxidants. In particular, tyrosine has a benzene ring highly activated by a very strong electron-donating hydroxyl (OH), and therefore, it is vulnerable to attack by an oxidant.

Amino acids with sulfur atoms in side chains, such as cysteine and methionine, are also easily oxidized. This is because the sulfur atom not only has two lone electron pairs, but also is relatively large, which makes it easy to oxidize.

To some degree, amino acids with nitrogen atoms in their side chains, such as histidine, lysine and arginine, are readily oxidized. This is because the nitrogen atom has one lone electron pair, so these amino acids can also be attacked by oxidants.

Amino acids with stable groups and atoms (such as alkyl group, phenyl group) in their side chains are inert to oxidizing agents, exhibiting lower or no antioxidative capability. Included in this category are glycine, alanine, valine, leucine, isoleucine, threonine, serine, aspartate, glutamate, asparagine, glutamine, phenylalanine and proline.

Although antioxidant activity could be predicted based on amino acid structures, further testing may help to elucidate more accurate trends, as well as nonlinear relationships [25]. The two methods used in this paper are widely accepted for the determination of antioxidative activity; the principles involved are different. The micro-potassium permanganate method depends on the fact that potassium permanganate is a strong oxidant [26] and that it can react with reducing agents to generate brown hydrated manganese dioxide in weak acidic, neutral or alkaline solutions. The iodometric method is based on the principle that the standard electrode potential of the I_2_/I^−^ pair is neither high nor low, so iodine can act as either an oxidizing or reducing agent, depending on the conditions [27,28]. We wish to determine if the two methods confirm each other, in order to obtain reliable results. The antioxidative ability of amino acids and their relative intensities cannot be assessed with accuracy, so it is necessary to perform systematic experiments and make a quantitative comparison. The results from the two methods indicate that they are highly consistent for most amino acids, but for a few, the methods display some inconsistency from the different ways according to different principles. We have therefore chosen the product of experimental values obtained from the two methods as an evaluating index of AOC for amino acids. The experimental results are largely in accordance with theoretical analysis of the antioxidative ability of these amino acids.

The limitation of this study is that AOCs should be compared between amino acids and other antioxidants. However, our initial thinking was just to classify the 20 alpha-amino acids based on the total antioxidant capacity, and we compared in different ways and determined to choose a relative one to arrive at our purpose. We will do further research on this topic in the near future.

## 5. Conclusions

Twenty amino acids could roughly be classified into two categories according to their antioxidative capacity. Seven amino acids, including tryptophan, methionine, histidine, lysine, cysteine, arginine and tyrosine, called antioxidative amino acids, were greater in total antioxidative capacity than the other 13 amino acids.

## Figures and Tables

**Table 1:** Molecular weights of amino acids.

Amino Acid	Molecular Weight	Amino Acid	Molecular Weight	Amino Acid	Molecular Weight
Alanine	89.09	Glycine	75.07	Proline	115.13
Arginine	174.20	Histidine	155.15	Serine	105.09
Asparagine	132.12	Phenylalanine	165.19	Threonine	119.12
Aspartic acid	133.10	Isoleucine	131.17	Tryptophan	204.23
Cysteine	121.16	Leucine	131.17	Tyrosine	181.19
Valine	117.15	Lysine	146.19	Glutamine	146.14
Glutamic acid	147.13	Methionine	149.21	-	-

**Table 2:** Comparison of the antioxidant activity of amino acids based on experimental results.

Amino Acid	Antioxidant Activity			Amino Acid	Antioxidant Activity		
	AUC-Mn	AUC-I	Total		AUC-Mn	AUC-I	Total
Tryptophan	0.953	0.057	0.054	Valine	3.551	2.121	7.532
Methionine	1.151	0.049	0.056	Serine	3.397	2.232	7.582
Histidine	1.198	0.056	0.067	Glycine	3.572	2.177	7.776
Lysine	1.931	0.158	0.305	Glutamine	3.556	2.198	7.816
Cysteine	1.851	0.198	0.366	Threonine	3.507	2.297	8.056
Arginine	2.969	0.289	0.858	Asparagine	3.598	2.257	8.121
Tyrosine	1.146	1.324	1.517	Isoleucine	3.596	2.334	8.393
Proline	3.202	2.188	7.006	Glutamic	3.584	2.498	8.953
Alanine	3.558	1.972	7.016	Aspartic	3.773	2.475	9.338
Phenylalanine	3.471	2.107	7.313	r (*p*)	0.869 (<0.001)		
Leucine	3.530	2.125	7.501	Blank control	3.610	2.381	8.595

AUC-Mn: the area under the curve for the potassium permanganate method; AUC-I: the area under the curve for the iodometric method; Total: total antioxidant activity (product of the experimental data obtained from the two methods).
